# The Influence of Antral Ulcers on Intramural Gastric Nerve Projections Supplying the Pyloric Sphincter in the Pig (*Sus scrofa domestica*)—Neuronal Tracing Studies

**DOI:** 10.1371/journal.pone.0126958

**Published:** 2015-05-11

**Authors:** Michal Zalecki

**Affiliations:** Department of Animal Anatomy, Faculty of Veterinary Medicine, University of Warmia and Mazury, Olsztyn, Poland; Federal University of Rio de Janeiro, BRAZIL

## Abstract

**Background:**

Gastric ulcerations in the region of antrum pylori represent a serious medical problem in humans and animals. Such localization of ulcers can influence the intrinsic descending nerve supply to the pyloric sphincter. The pyloric function is precisely regulated by intrinsic and extrinsic nerves. Impaired neural regulation could result in pyloric sphincter dysfunction and gastric emptying malfunction. The aim of the study was to determine the effect of gastric antral ulcerations on the density and distribution of intramural gastric descending neurons supplying the pyloric sphincter in pigs.

**Methodology/Principal Findings:**

The experiment was performed on 2 groups of pigs: healthy gilts (n=6) and gilts with experimentally induced peptic ulcers in the region of antrum pylori (n=6). Gastric neurons supplying pyloric sphincter were labeled using the retrograde neuronal tracing technique (20μl of Fast Blue tracer injected into the pyloric sphincter muscle). After a week survival period the animals were sacrificed and the stomachs were collected. Then, the stomach wall was cross-cut into 0.5cm thick sections taken in specified intervals (section I - 1.5cm; section II - 3.5cm; section III - 5.5cm; section IV – 7.5cm) starting from the sphincter. Consecutive microscopic slices prepared from each section were analyzed under fluorescent microscope to count traced neurons. Obtained data were statistically analyzed. The total number of FB-positive perikarya observed within all studied sections significantly decreased from 903.3 ± 130.7 in control to 243.8 ± 67.3 in experimental animals. In healthy pigs 76.1 ± 6.7% of labeled neurons were observed within the section I, 23.53 ± 6.5% in section II and only occasional cells in section III. In experimental animals, as many as 93.8 ± 2.1% of labeled cells were observed within the section I and only 6.2 ± 2.2% in section II, while section III was devoid of such neurons. There were no traced perikarya in section IV observed in both groups of pigs.

**Conclusions/Significance:**

Obtained results revealed for the first time significant impact of antral ulcerations on intramural descending nerve pathways supplying the pyloric sphincter in pigs, animals of increasing value in biomedical research and great economic importance.

## Introduction

Gastric ulceration is a disorder known for many years in both, humans and animals. Despite the years of studies there are still many queries in this subject. Starting from etiology, which seems to be a multiple process [1], through the methods of diagnosis, treatment and even the classification of ulcers there was no full consistency between scientists and our knowledge has changed over the years of studies. A well-established experimental method, termed “acetic acid ulcer model”, is commonly used in a broad spectrum of research on gastrointestinal ulcers in many species [2]. The ulcers may vary in size of diameter, depth of penetration and localization within the gastrointestinal tract. Large ulcer diameter seems to be correlated with hindered healing process [3] and the risk of gastric cancer development [4–6]. Ulcers localized in the close proximity to the pyloric sphincter have been clinically associated with a characteristic “pyloric syndrome” complex [7], which clinical signs differed significantly from symptoms associated with other ulcer locations [8]. Gastric emptying was delayed only in patients with distal gastric ulcers, while proximal gastric or duodenal ulcers accelerated this process [9]. Such delay could even lead to the symptoms resembling adult hypertrophic pyloric stenosis (AHPS) [10]. Many studies performed in humans have suggested the pyloric sphincter problems, as: pyloric dysfunction [11], narrowing of the pyloric ring and abnormalities in the thickness of pyloric musculature [12,13] in patients with gastric ulcerations. The pyloric sphincter function has to be precisely regulated by intrinsic and extrinsic nerves [14–19] to ensure the proper outflow of the gastric contents. Intrinsic neurons, that compose enteric nervous system, are enormously important in gastrointestinal motility regulation [20,21]. An intact intramural descending neural pathways have been shown to play a crucial role in physiological gastric emptying process [22–25]. Disturbances in the sphincter muscles innervation may affect the function of the pyloric orifice which disrupts the function of the lower gastrointestinal tract. Pyloric dysfunction may lead to many other gastrointestinal problems as maldigestion, malabsorption and malnutrition [26]. Gastric ulcerations localized in the distal part of the stomach are commonly observed in many mammals and humans. Such localization of ulcers may suggest their influence on gastric intramural descending neuronal pathways supplying the sphincteric muscles. Although there are many articles focused solely on the gastric ulcerations or the pyloric disorders, there are no publications linking both issues in the context of the nervous system. It seems to be extremely interesting question, whether the antral ulcers disturb the intramural descending neural pathways, which can directly influence the pyloric sphincter activity. Knowledge on the gastrointestinal tract of the pig, an animal of a great economic value and increasing importance in biomedical research [27–30] seems to be of particular interest. Moreover, the swine is an omnivorous animal, which makes it especially valuable model for research on the digestive tract, in the context of human disorders. Therefore, the aim of the present study was to establish the changes in the number and distribution of intramural descending neurons supplying pyloric sphincter in pigs with experimentally induced ulcers in the distal part of the stomach.

## Materials and Methods

The handling of animals and all experimental procedures were in accordance with the rules of the Local Ethics Committee of the University of Warmia and Mazury in Olsztyn (permit number 03/2012) affiliated to the National Ethics Commission for animal experimentation (Polish Ministry of Science and Higher Education). All surgery was performed under sodium pentobarbital anesthesia, and all efforts were made to minimize suffering.

**Fig 1:**
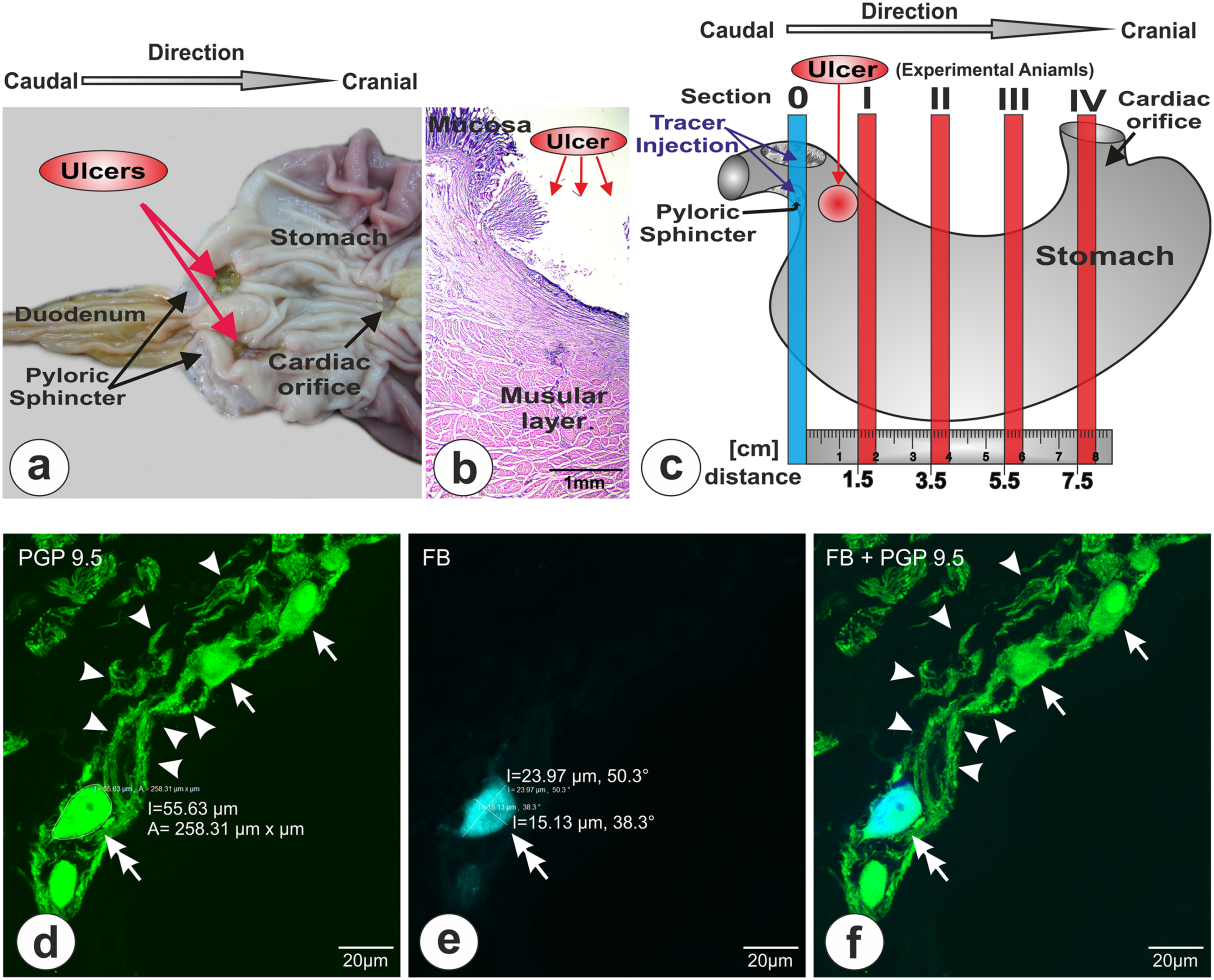
Tissue sampling and cell measurements. (**a**) Picture presenting the interior surface of the "experimental animal" stomach, exposed by cutting along the greater curvature of the stomach. Ulcers are indicated by red arrows. (**b**) Microphotograph showing Haematoxylin and Eosin staining of the gastric ulcer margin. The gastric mucosa shows a defect (red ellipse with the inscription “Ulcer” and arrows) that extends into the deep muscular layers. (**c**) Diagram showing the method of tissue sampling. The tracer injection site is indicated by the violet arrows. The red vertical stripes symbolize the subsequent 0.5 cm thick transverse sections taken in specific intervals (section I—1.5 cm; section II—3.5cm; section III—5.5 cm; section IV—7.5cm) starting from the pyloric sphincter (section 0—blue vertical stripe). Subsequently, each of these sections (I, II, III, IV) was cut into 20 μm thick cryostat consecutive microscopic slices for future processing. The red circle symbolizes the localization of ulcer induced in experimental animals. (**d, e, f**) Photomicrographs showing: (**d**) bundles of PGP-immunoreactive fibers (arrowheads) and perikarya (arrows). Double arrow points to the perikaryon which was fast blue (FB) positive [the perimeter (l) and area (A) of the cell body are included]; (**e**) fast blue (FB) positive cell body (double arrow) with lines indicating its dimensions; (**f**) the image formed by merging both channels (green: PGP 9.5 and blue: FB).

Twelve sexually immature gilts of the Polish Large White breed (body weight approx. 20 kg) obtained from commercial fattening farm were used in the experiment. Animals were divided into control (n = 6) and experimental (n = 6) groups. Thirty minutes before the main anesthetic the animals of both groups were pre-treated with azaperone (Stresnil, Janssen Pharmaceutica, Belgium, 0.4 mg/kg b.w., i.m.) and atropine (Polfa, Poland, 0.04 mg/kg b.w., s.c.). Then, the animals were generally anaesthetized with pentobarbital (Vetbutal, Biowet, Poland, 30 mg/kg b.w.) given intravenously. During the whole surgery the breathing and heartbeat were monitored. Next, the pylorus was exposed via midline laparotomy and 20 μl of 5% aqueous suspension of fluorescent tracer Fast Blue (Polysciences, Inc; Cat# 17740) was injected with a Hamilton microsyringe into 4 places (about 5μl into every place) arranged around circumference of the pyloric sphincter wall (as described in detail previously [17]). In the group of experimental animals an additional bilateral injections of 40% acetic acid solution were performed to produce gastric ulcers (according to the acetic acid ulcer models procedure [2]). After exposing the stomach, 1 cm^3^ of acid solution was injected with a single-use insulin micro-syringe (Micro-Fine Plus, Becton, Dickinson and Company, USA) into the submucosal layer of the anterior and posterior wall of the antrum, about 1.5 cm from the pyloric orifice. The length of the insulin micro-syringe needle (8 mm) utilized in the study (in relation to the thickness of the stomach wall in the place of acid deposition) ensured the accuracy of the injection depth. A sterile tampon was tightly placed on the inserted needle at the time of injection and maintained at the site for about 30 s after the needle removal to avid solution leakage. A weal-like swelling in the place of injection confirmed the accuracy of the injection site. Finally, the midline abdomen incision wound was sutured and secured by antibiotic (chlortetracycline, Animedazon Spray, aniMedica Gmbh, Germany) and micronized aluminum (Alu Spray, Arendonk, Belgium) spraying. Following the recovery from anesthesia the antibiotic (Betamox L.A., ScanVet, Poland, 15 mg/kg b.w., i.m.) and meloxicam (Metacam, Boehringer Ingelheim Vetmedica GmbH, Germany, 0.4 mg/kg b.w., i.m.) injections were performed. Finally the animals were moved to individual pens with unlimited access to water. On the next day after surgery, the animals have unlimited access to the feed. To minimize the pain and suffering the meloxicam (Metacam, Boehringer Ingelheim Vetmedica GmbH, Germany, 0.4 mg/kg b.w., i.m.) injections were performed every 24 hours. The condition of animals was monitored at least 5 times a day. After a week the animals were again deeply anaesthetized (as described previously) and transcardially perfused with a 4% solution of paraformaldehyde in 0.1M phosphate buffer (pH 7.4). Subsequent to perfusions the stomachs were removed and cut into anterior and posterior part along the greater and lesser curvatures. In experimental animals peptic ulcers were observed in both parts, about 0.5–1 cm from the pyloric orifice (Fig 1a). The ulcers penetrated deeply into the muscular layer (Fig 1b). The diameter of ulcers ranged between 0.5 till 1.6 cm. To standardize results obtained only animals with ulcer diameter equal to or greater than 1 cm were analyzed (n = 5). All the gastric parts were thoroughly washed in PBS to remove food debris located on the mucosa. Next, the tissues were post—fixed in the same fixative as used for the perfusion (60 min., temperature of fixative + 4 C°), rinsed in PBS for 2 days and transferred to and stored in 18% buffered (pH 7.4) sucrose solution for 3 weeks. Then, each part of the stomach wall (anterior and posterior) was cut into 0.5 cm thick transverse sections taken in specific intervals (section I—1.5 cm; section II—3.5cm; section III—5.5 cm; section IV—7.5cm) starting from the pyloric sphincter (Fig 1c). Finally, 20 μm thick cryostat consecutive microscopic slices of all collected sections (I, II, III, IV) were prepared and mounted on chrome alum—gelatin—coated slides, air—dried and stored desiccated at -23°C until processing.

Microscopic tissue slides from each section (I, II, III, IV) were analyzed under fluorescent microscope (Axiophot, Zeiss, Germany) equipped with a filter set suitable for observation of FB to localize and count neurons containing the tracer. Calculations of the number of traced perikarya were made in every fourth tissue strip in order to obtain a distance greater than the dimensions of the cells. The size of traced cells was determined by confocal laser microscopy “z-stack” function and 3D software measurements (including measurements along the z axis) (Zen 2009, ver. 5.5.0.282 and LSM Image Browser, ver. 4.02, Zeiss) at consecutive sections immunostained with primary antibody against pan neuronal marker PGP 9.5 (mouse anti-PGP 9.5, dilution 1:600, code 7863–2004, AbD Serotec) and corresponding secondary antibody (AlexaFluor 488, goat anti-mouse, dilution 1:500, code A11001, Invitrogen, USA) (Fig 1d–1f). All staining procedures and controls were performed according to the previously described protocol [17]. The cell measurements were performed at a group of 30 FB/PGP-positive neurons. Cell dimensions of the cross-sectional cutting plane were measured at the level of the nucleus, perpendicular and longitudinal to the longest axis of the perikaryon (Fig 1e). None of the horizontal nor vertical cell body dimensions exceeded 45 μm. This has provided certainty that none of the FB-positive cell body was counted twice (as described previously [17–19]). Finally, the slides were photographed with a confocal laser microscope (LSM 700, Zeiss). The results were statistically analyzed using GraphPad Prism software (ver. 6.05, GraphPad Software Inc., USA) and presented as a mean ± standard error (SEM). In order to verify the ratio of traced cells between the individual sections (I, II, III, IV), regardless the varying number of traced cells in the individual stomachs, the data obtained from each section were additionally presented as percentages. The differences in the number of cells observed between control and experimental animals were analyzed by the Student *t*-test, and considered to be significant at P < 0.05.

The histochemical Haematoxylin and Eosin staining of the ulcer was carried out according to Ehrlich (Fluka; code 03972) and the slides were photographed by use of stereo microscope (SteREO Discovery V8, Zeiss) equipped with the camera (MotiCam 2500).

## Results

**Fig 2:**
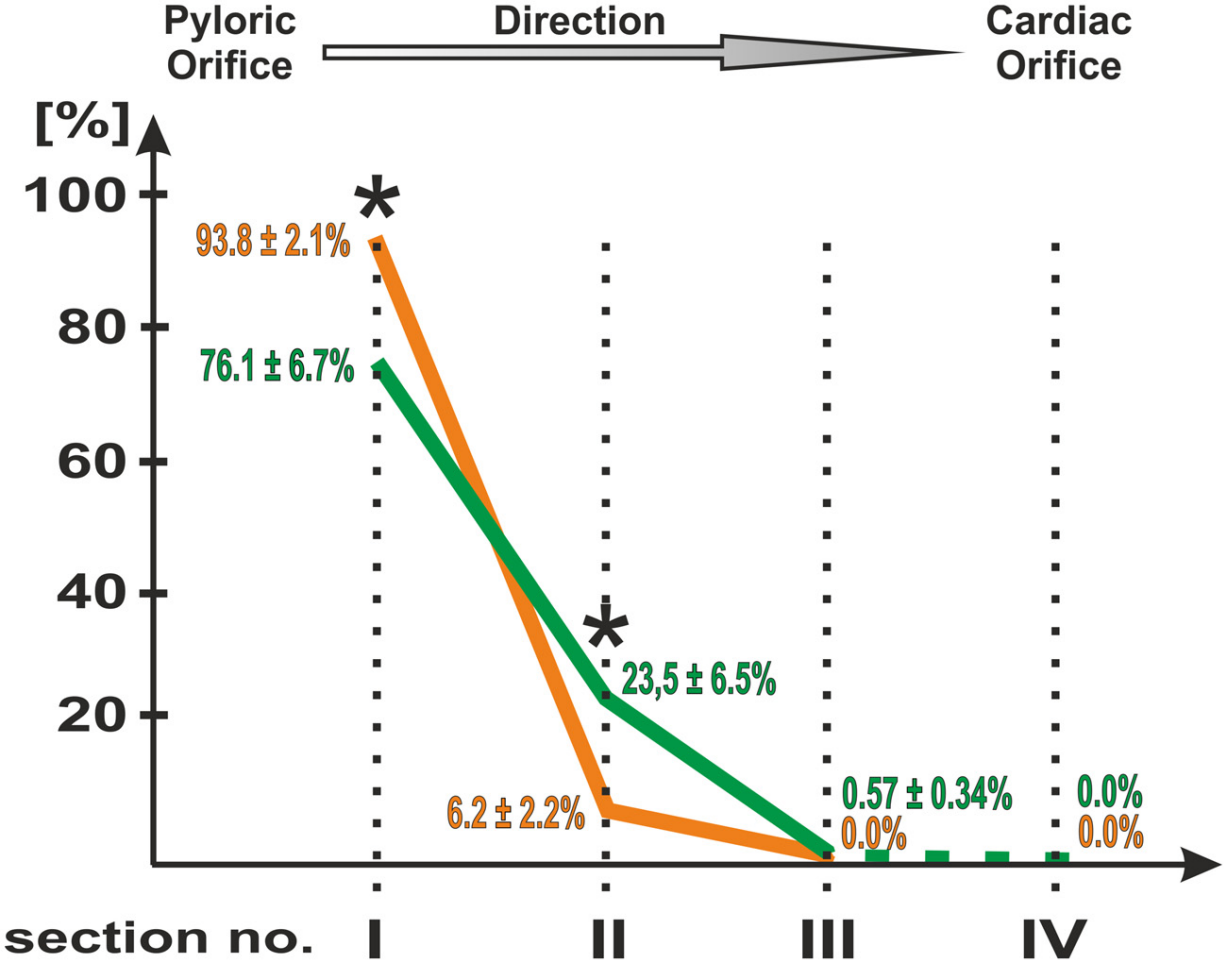
Graph presenting percentage of FB-positive neurons in studied sections. Percentage of FB-positive neurons observed in subsequent sections (I, II, III, IV) in control (green) and experimental (orange) animals. Observed intergroup differences were statistically significant (P<0.05) in section I and II (asterisk).

**Fig 3:**
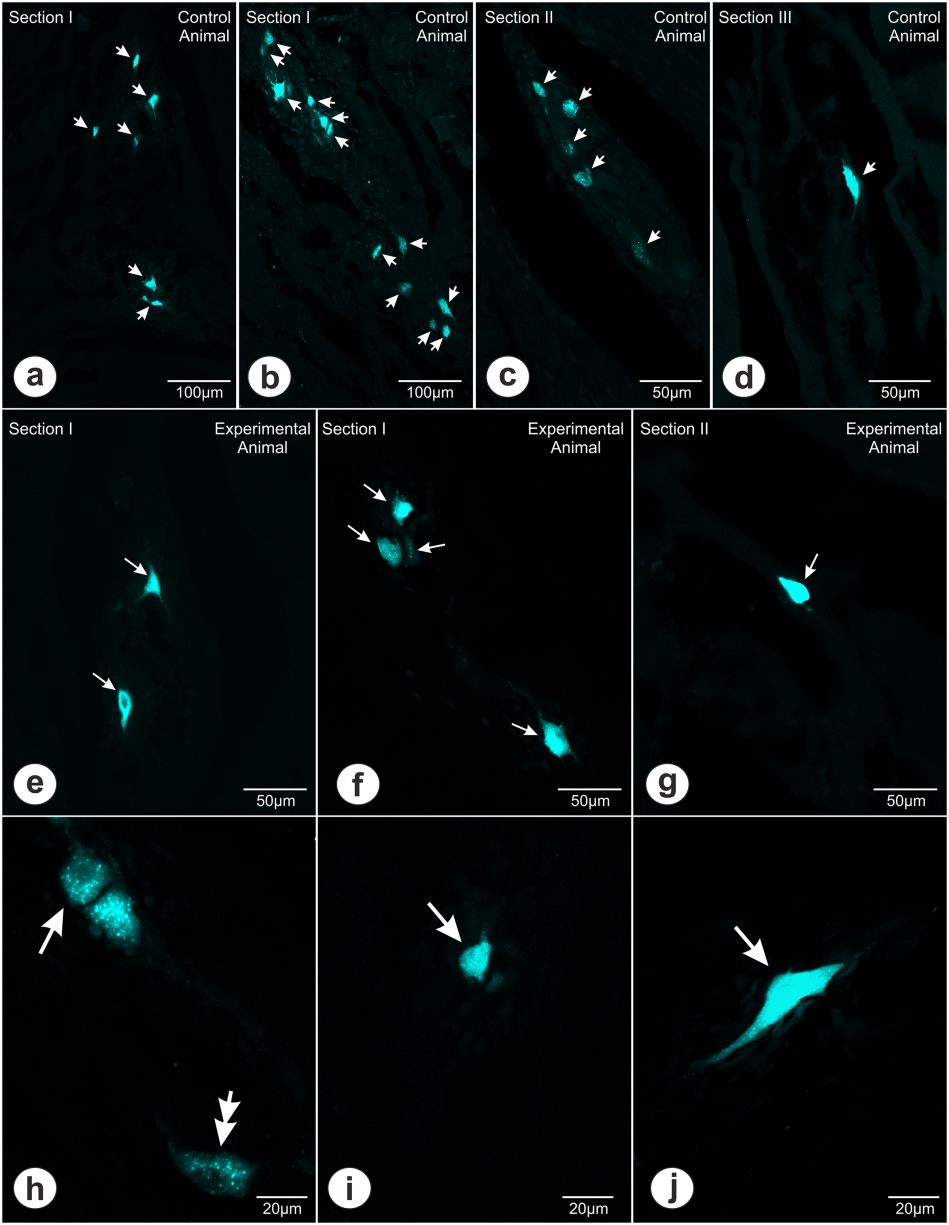
Traced neurons observed in studied sections. (**a, b, c, d**) Microphotographs presenting the Fast Blue positive neurons (arrows) in section I (**a, b**), II (**c**) and III (**d**) obtained from the stomachs of control animals. In section I the traced neurons occurred mostly in groups of 4–7 neurons (**a**), although there were more numerous clusters, up to 12 cells observed (**b**). In section II the traced cells were observed in groups up to 5 neurons (**c**), while in section III only occasional cells were noticed (**d**). There were no traced neurons observed within section IV. (**e, f, g**) Photomicrographs showing the Fast Blue positive neurons (arrows) in section I (**e, f**), and II (**g**) obtained from the stomachs of experimental animals. In section I the majority of labeled cells was scattered within the myenteric ganglia singly or in pairs (**e**), however, occasional groups up to 4 labeled neurons were additionally noticed (**f**). In section II the traced cells were observed mostly singly (**g**). There were no labeled neurons observed within section III nor IV. (**h, i, j**) Higher magnification microphotographs showing the Fast Blue labeled perikarya (arrows) of different sizes and shapes: (**h**) medium-sized oval (single arrow) and round (double arrow), (**i**) small-sized round and (**j**) large-sized triangular in shape labeled perikarya.

**Fig 4:**
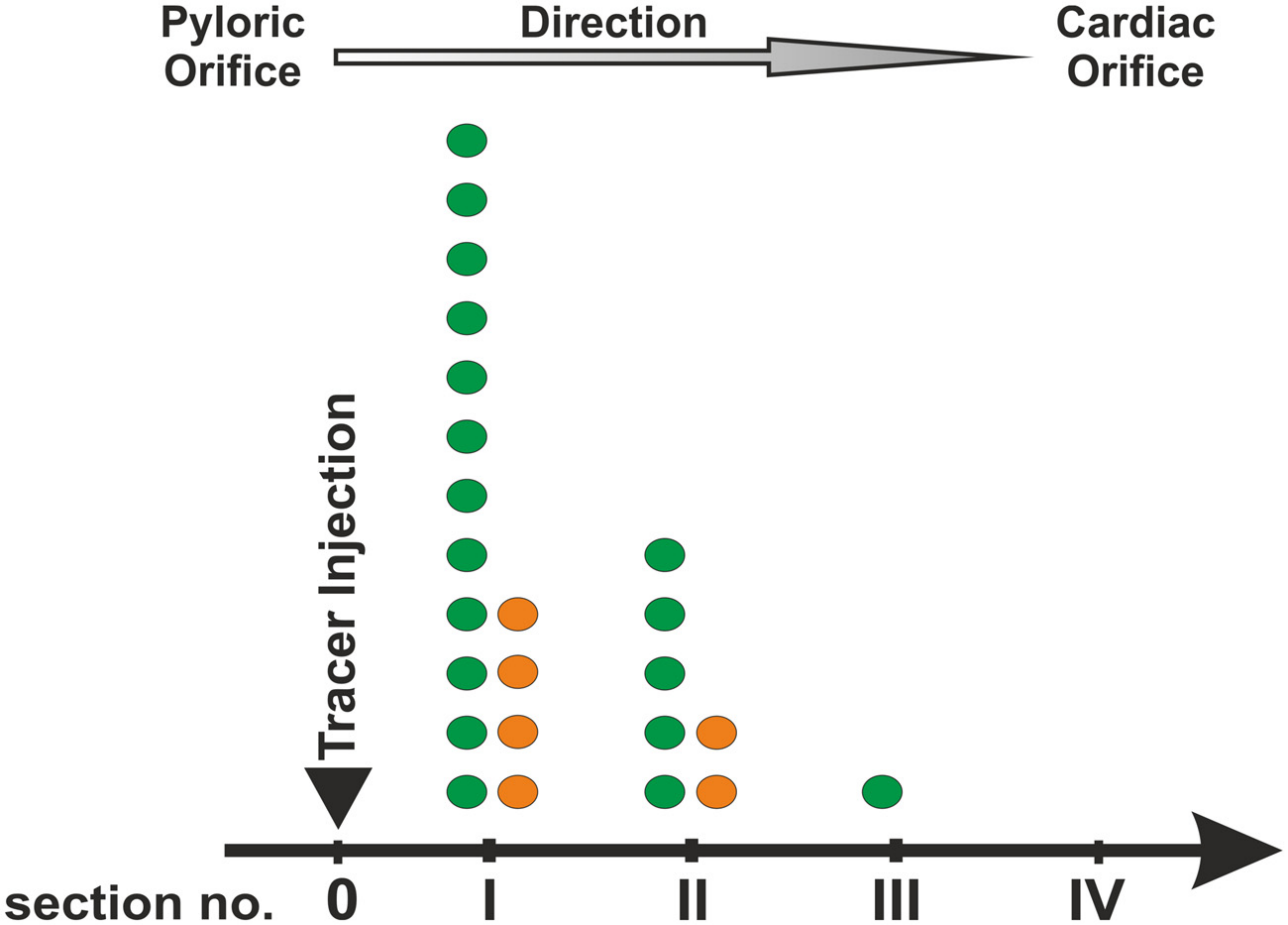
Histogram showing the maximum number of traced neurons in studied sections.

All retrogradely labeled neurons were observed within the myenteric plexus. The total number of FB positive cells observed within all studied sections in control group was 903.3 ± 130.7 per animal, while in experimental group 243.8 ± 67.3 per animal and the existing intergroup difference was statistically significant (P<0.05). In control animals the traced neurons were dispersed throughout the section I, II, and III while in experimental animals the section III was devoid of such cells (Fig 2). In both groups of animals there were no traced neurons observed in section IV. The number of labeled cells observed within the individual sections decreased rostrally, towards the cardia of the stomach. In control animals 76.1 ± 6.7% of labeled neurons were observed within the section I (1.5 cm from the pyloric orifice), 23.53 ± 6.5% within the section II (3.5 cm from the pyloric orifice), and only occasional cells (0.57 ± 0.34%) in section III (4.5 cm from the pyloric orifice) (Fig 2). In experimental animals, as many as 93.8 ± 2.1% of labeled cells were observed within the section I and only 6.2 ± 2.2% in section II (Fig 2). Observed intergroup differences were statistically significant (P<0.05). The arrangement of traced cells within the myenteric ganglia changed depending upon their distance from the pyloric orifice—in section I, obtained from control animals, the traced neurons occurred mostly in groups of 4–7 neurons (Fig 3a), although there were more numerous clusters, up to 12 cells (Figs 3b and 4), noticed. In section II traced cells were observed in groups up to 5 neurons (Figs 3c and 4), however majority of cells occurred solely. Occasional FB-positive perikarya observed within the section III were arranged individually (Figs 3d and 4). In experimental animals, the vast majority of labeled cells observed in section I and II were scattered within the myenteric ganglia in pairs (Fig 3e) or singly (Fig 3g). However, in section I FB-positive neurons occasionally gathered in groups up to 4 perikarya (Figs 3f and 4).

The maximum number of FB labeled perikarya (dots) observed in individual myenteric ganglia within subsequent sections (I, II, III, IV) in control (green) and experimental (orange) animals.

The vast majority of Fast Blue labeled neuronal cell bodies observed in both groups of animals were oval or round in shape (Fig 3h) and measured about 15.3 ± 0.5 x 22.6 ± 0.8 μm in diameter. Occasionally, smaller (about 10 μm) (Fig 3i) or larger (up to 45 μm) triangular (Fig 3j) or multipolar in shape perikarya were noticed. The intensity of FB-fluorescence varied from moderate (Fig 3h) to strong (Fig 3j).

## Discussion

Presented study has demonstrated for the first time a significant impact of antral ulcerations on intramural descending nerve pathways supplying the pyloric sphincter muscles in the pig. Results obtained clearly indicate that gastric ulcers localized proximally to the pyloric sphincter significantly reduce the number of intrinsic gastric neurons projecting to the pylorus as well as change the distribution of such neurons within the consecutive sections of the gastric wall. The enteric nervous system and intrinsic neuronal pathways have been well demonstrated to play a crucial role in the regulation of gastrointestinal tract motility [14–16]. The impaired gastric motility in the distal stomach and problems with gastric emptying were described in patients with gastrointestinal ulcerations since the forties of the last century [31–35]. Some of the authors presumed that such symptoms may have been related to the disturbances in the gastric innervation and interruption between action potentials and smooth muscles [35]. Although some studies have analyzed the histological changes within the gastric wall of the patients with ulcers, only a few noted the abnormalities and reductions in the number of Auerbach’s plexuses and corresponding nerve cells [13,36]. However, none of the existing studies have analyzed the changes exactly in the descending gastric neurons supplying the pyloric sphincter. The importance of the intramural descending gastric neural pathways in the regulation of the pyloric sphincter function and gastric emptying has been clearly demonstrated in experiments performed in many species [22,24,25,37–39]. Most of the authors have indicated that descending gastric pathways to the pylorus were inhibitory in nature and led to the relaxation of the sphincteric muscles [40].

One of the criteria on the basis of which gastric ulcers might be classified is their anatomical localization in relation to the pyloric sphincter. All the researches have distinguished ulcers localized near the pylorus, although they have used different nomenclature for such ulcers: “Type I, Subtype 1: prepyloric ulcers” [41–43]; ulcers of “prepyloric antrum” [44]; “antral, prepyloric” or even “pyloric channel” ulcers [45]. It should be emphasized that only ulcers localized in the pyloric antrum produced consistent delay in gastric emptying, while proximal gastric or duodenal ulcer locations accelerated gastric outflow. Such specific symptoms have been described as a “pyloric syndrome complex” already in the sixties of the last century [7]. It seems to be reasonable to assume that disturbances in descending gastric nerve pathways might have contributed to such specific phenomenon.

The reduced number of descending “pyloric” neurons observed in the present experiment in animals with antrum ulcers seems to be consistent with such assumption and results indicating the gastric outflow problems in patients with antral ulcerations [7–9].

The ulcers induced in the present study were large in diameter and deeply penetrated into the muscular layer. According to some authors, the large size of gastric ulcer was closely related with difficulties in its treatment [3], the likelihood of ulcer malignancy [6,46,4] and lymph node metastasis [5,47]. Extensive, deeply penetrating ulcers destroy deeper tissues of the gastric wall, including nerve pathways. Such pathological conditions occurring within the gastric wall are likely to influence the gastric emptying control. Correspondingly, experimental antral nerve pathway transections performed in dogs [25] and pigs [22] significantly retarded gastric emptying. The partial destruction of myenteric plexus with benzalkonium chloride (BAC) in rats produced a consistent delay in gastric emptying [48]. All these physiological observations seem to be consistent with neuroanatomical results obtained in the present experiment.

Similarly to human medicine, gastrointestinal ulcerations are quite significant problem in veterinary. For the porcine species, gastric ulceration is a common disorder, mostly affecting grower-finisher pigs, which results in a huge economic loss [49]. Bleeding gastric ulcers are one of the most common cause of the mortality in animals from 3 to 6 months of age [50], while animals with severe ulcers are likely to grow more slowly than the healthy ones [51].

Results obtained in the present study have demonstrated for the first time the destructive effect of the large ulcerations on the descending neural pathways innervating the pyloric sphincter muscles. The redistribution and reduction in the number of gastric descending nerve cells supplying the pyloric muscles seem to give some neuroanatomical explanation of the reasons of gastric emptying problems in patients with antral ulcerations. However, the disturbances in the tissue continuity occurring due to the fact of ulcer lesions in the gastric antrum could additionally influence the motility of the whole distal part of the stomach. It seems to be reasonable to conclude that the sum of all pathological factors occurring in mammals with gastric ulcerations disturbs the flow of gastric chyme downstream and finally results in gastric emptying problems. In the view of the presented results, future studies focused on the biologically active substances involved in the intrinsic neuronal control of the pyloric sphincter in ulcer disease seem to be of particular interest.
